# Accelerating perennial crop improvement via multi‐omics‐based predictive breeding

**DOI:** 10.1002/tpg2.70058

**Published:** 2025-11-18

**Authors:** Hannah Robinson, Carlos A. Robles‐Zazueta, Kai P. Voss‐Fels

**Affiliations:** ^1^ Department of Plant Breeding Hochschule Geisenheim University Geisenheim Germany

## Abstract

Perennial crops are positioned at a critical juncture, facing intensifying environmental challenges that threaten productivity. Despite the high value of these crops, breeding gains in perennials are notably slow due to prolonged breeding cycles, often exceeding several decades, and thereby limiting their capacity to adapt to increasing climatic stressors. In contrast, annual crops have begun to leverage predictive breeding methods to incorporate multi‐omics data, paving the way for a new era of accelerated genetic improvement. Multi‐omics approaches integrate diverse datasets, ranging from genomic to proteomic layers, and likely more comprehensively capturing system features of regulatory networks that link the genome and phenotype. In this review, we assess the current landscape of predictive breeding in perennials by examining single‐omic approaches alongside emerging omics resources, and we compare these trends with established multi‐omics‐based prediction frameworks in annual crops that have yielded enhanced predictive ability and novel biological insights. Building on these comparisons, we outline key considerations for implementing multi‐omics‐based genetic improvement frameworks in perennials, emphasizing the need for an end‐to‐end, reproducible, and scalable system that integrates multidimensional datasets and models both additive and nonadditive genetic effects across genotype‐by‐environment‐by‐management interactions. We also address significant challenges, including high data dimensionality, complex genotype‐by‐environment interactions, and limited training population sizes, and propose cross‐institutional collaborations to pool resources, as well as the use of breeding program simulation tools to optimize multi‐omics integration into practical breeding strategies. Despite current limitations, multi‐omics‐based predictive breeding holds great promise as a powerful tool for rapid genetic improvement in perennial crops.

## Core Ideas

- Multi‐omics prediction captures interactions across multiple biological layers, linking the genome to the phenotype.
- Multi‐omics‐based predictive breeding holds potential for accelerating genetic gain in perennial crops.
- Key challenges for predictive breeding in perennials include high data complexity, genotype‐by‐environment interaction (GEIs), and constrained resources.
- Cross‐institutional collaborations and use of breeding program simulation tools may optimize integration strategies.

## Plain Language Summary

Perennial crops are increasingly threatened by climate change, and their adaptation relies on genetic improvement. However, breeding perennials has been slow due to long breeding cycles often spanning decades. In contrast, annual crops benefit from predictive breeding techniques that integrate multi‐omics data, capturing multiple layers of biological information to accelerate genetic improvements. This review examines the application of multi‐omics in annual crop breeding and explores how similar methods could be adapted for perennials to achieve faster genetic gains. Key challenges for perennials include managing complex datasets, modeling gene–environment–management interactions, and overcoming significant resource constraints. We propose that enhanced collaboration and targeted simulation tools can address these issues. Multi‐omics prediction holds great potential for enhancing perennial crop breeding, enabling faster and more efficient genetic improvement.

**Abbreviations**
ANN: artificial neural network
CGM–WGP: crop growth model–whole genome prediction
CV: cross‐validation
FA: factor analytic
GBLUP: genomic best linear unbiased prediction
GEI: genotype‐by‐environment interaction
GS: genomic selection
IFA‐LMM: integrated factor analytic‐linear mixed model
NIR: near infrared reflectance
PS: phenomic selection
SNP: single nucleotide polymorphism
TP: training population
TPE: target population environments

## 1 INTRODUCTION

While the increasing demands of the changing agricultural landscapes coupled with the need for increased productivity challenge all crop systems, perennial crops seem particularly vulnerable. Perennials continuously regenerate, delivering sustained yields without replanting, and generally impart fewer environmental impacts (e.g., less soil erosion), compared to annual crops. However, this regenerative mechanism also increases their susceptibility to environmental stressors, and as such with the changing climate, their production may become less sustainable, necessitating prompt adaptation measures. Genetic improvement through traditional breeding remains one of the most viable and sustainable solutions (Xu et al., 2017). However, traditional breeding faces a critical limitation in that it is exceedingly slow. For many perennials like grapevine and various Rosaceae species, the average breeding cycle can span up to, or even exceed, 25 years, severely limiting the rate of genetic gain.

In recent years, breeding research has advanced considerably, evolving into a highly data‐driven discipline centered on predictive methodologies. Predictive breeding, which leverages integrative data alongside advanced computational tools and modeling approaches, offers great potential to accelerate genetic improvement. The range of interdisciplinary data available for predictive breeding has recently expanded due to cost reductions from technological advancements, and some common examples of such omics data are listed:

(1) Genomic: DNA sequence information, including nucleotide sequences, structural variants, and single nucleotide polymorphisms (SNPs).
(2) Epigenomic: Chemical modifications to DNA and histones, such as methylation and acetylation patterns.
(3) Transcriptomic: RNA sequence and abundance profiles, such as whole‐genome transcripts.
(4) Proteomic: Protein sequences, abundance, and/or modifications within a cell or tissue.
(5) Metabolomic: Concentration and identity of small‐molecule metabolites within a biological sample.
(6) Phenomic: Digital phenotyping profiles derived from spectral imaging and sensors.

One such example of the use of genomic data is genomic selection (GS), which leverages molecular markers and trained algorithms to predict trait performance without phenotyping (Meuwissen et al., 2001). The accuracy of GS models is influenced by factors such as training population (TP) size and genetic composition, trait complexity, and phenotypic data quality (Voss‐Fels et al., 2019).

Phenomic selection (PS) is a low‐cost, noninvasive, high‐throughput breeding method, which leverages high‐dimensional phenotypic data, often in the form of spectral reflectance profiles, to capture complex traits and their interactions with the environment (Rincent et al., 2018). These spectral profiles, also referred to as endophenotypes, can serve in place of or alongside molecular markers in GS, thereby defining genetic relatedness among individuals. PS is often considered cost‐effective because it relies on high‐throughput phenotyping tools, such as multispectral cameras or point sensors, which can be deployed on unmanned aerial vehicles, mobile platforms, or used in laboratory settings. This is feasible because a single sensor measurement captures a rich and complex data stream (e.g., spectral signatures, thermal patterns, 3D structures) simultaneously, allowing multiple distinct trait proxies including growth rates, canopy architecture, and stress responses to be computationally extracted from that same dataset at relevant spatial, temporal, and resolution scales.

**FIGURE 1:**
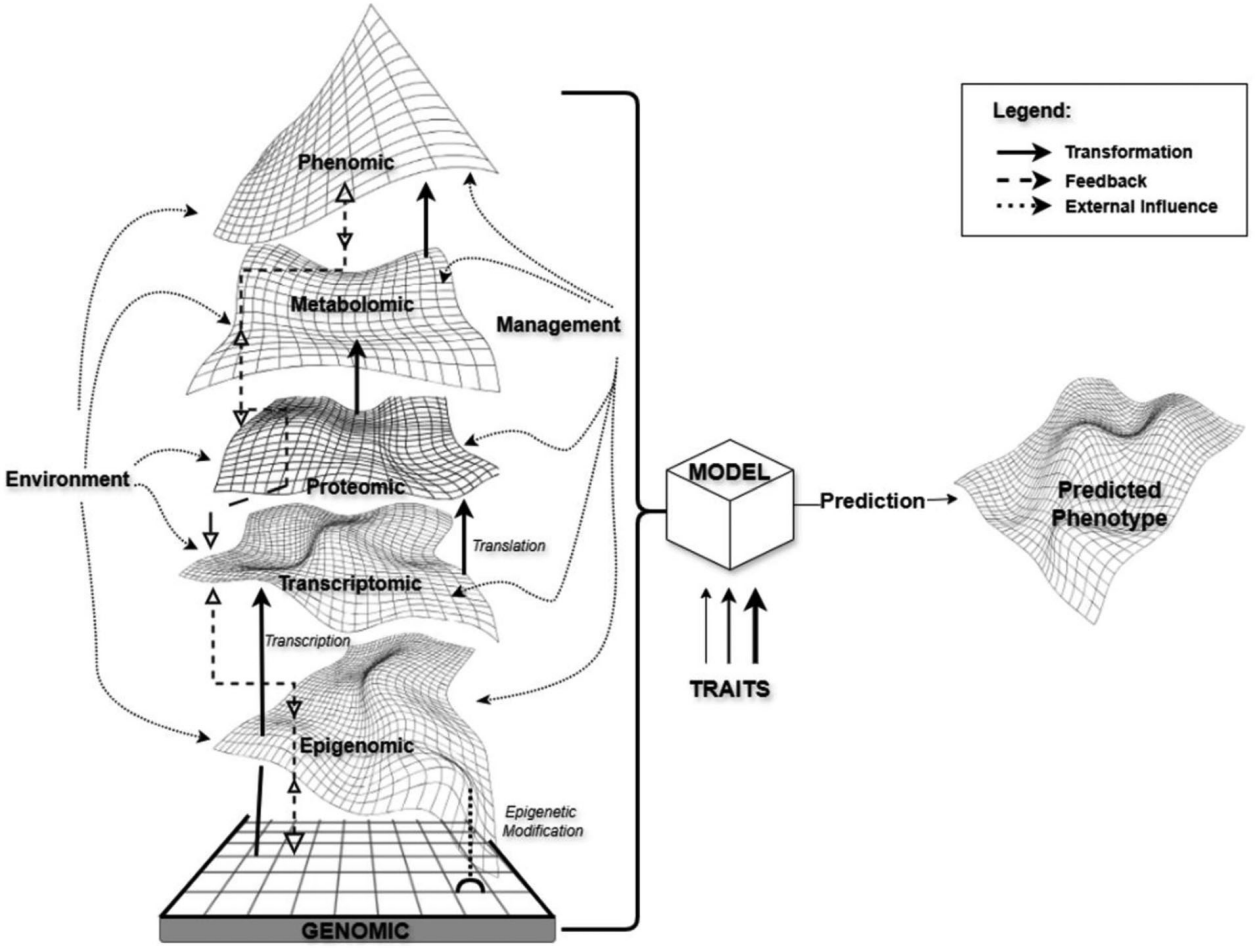
Conceptual framework for multi‐omic predictive modeling. The model integrates static genomic information (base grid) with data from progressively more dynamic intermediate layers. Thick bold arrows represent major transformation between omic layers (e.g., transcription, translation), dashed arrows demonstrate feedback between layers, and thin dotted arrows represent external environment and management interactions. External factors, which interact with and influence all biological levels, are co‐integrated with univariate or multivariate traits, with varying impact (depicted by arrow thickness), by the computational model (cube). This integration aims to capture the complex flow of information through multiple layers of biological regulation, resulting in an enhanced prediction of the final phenotype.

Multi‐omics prediction integrates two or more omics data sources to capture a broader range of molecular and biochemical processes influencing complex traits. This is a systems approach, where each data set represents distinct hierarchical and interactive layers between the genome and the phenotype, enabling the capture of intermediate regulatory and interactive networks both between omics layers and with the external environment and management practices (Rincent et al., 2018; Figure 1). To illustrate this further, the genome provides a stable, foundational layer of molecular information yet offers limited insight into dynamic biological processes. In contrast, downstream omics, such as the epigenome, transcriptome, proteome, metabolome, and phenome, respond to environmental cues and are likely to exhibit stronger and more dynamic responses compared to the relatively stable genome. For example, transgenerational epigenetic variation demonstrates how heritable changes in gene expression or cellular function, without alterations to the DNA sequence, can contribute to heritable variation for breeding (Kakoulidou et al., 2021). By integrating regulatory networks between the genome and phenotype, interrelationships among omics layers, and omics‐by‐environment‐by‐management interactions, multi‐omics prediction can enhance predictive ability and uncover deeper biological mechanisms. This approach is expected to support better informed selection and greater genetic gain.

While the promise of predictive breeding approaches is clear, the utilization in perennials remains limited. We assess this gap by reviewing the research landscape, first with a focus on single‐omic predictive breeding approaches covering their applications, challenges, and comparative merits. By drawing on examples from annual crops, we then outline emerging multi‐omics prediction frameworks and investigate the role, impact, and challenges of genotype‐by‐environment interaction (GEI) modeling within these contexts. Lastly, we delineate a conceptual, structured framework intended to facilitate the application of multi‐omics prediction within perennial crop breeding programs and address associated challenges and opportunities.

**Core Ideas**

- Multi‐omics prediction captures interactions across multiple biological layers, linking the genome to the phenotype.
- Multi‐omics‐based predictive breeding holds potential for accelerating genetic gain in perennial crops.
- Key challenges for predictive breeding in perennials include high data complexity, genotype‐by‐environment interaction (GEIs), and constrained resources.
- Cross‐institutional collaborations and use of breeding program simulation tools may optimize integration strategies.

## 2 FROM SINGLE‐OMICS TO MULTI‐OMICS‐BASED PREDICTION

### 2.1 Single‐omics approaches in predictive breeding of perennial crops

Despite the technology being first reported over two decades ago, GS is still in the early adoption phase for perennial crops. The foundational proof of concept research emerged over a decade ago in apple (Kumar et al., 2012), grapevine (Fodor et al., 2014), perennial ryegrass (Pembleton et al., 2018), and coffee (Sousa et al., 2019). Much of these early studies were focused on simulation of the technology or application in populations with highly related individuals, both of which have limitations for practical breeding application. However, the more recent GS studies have assessed several optimization approaches, such as combining related and unrelated populations to increase TP size (Minamikawa et al., 2024; Pembleton et al., 2018; Roth et al., 2020), variation in molecular marker density (Cazenave et al., 2022: Minamikawa et al., 2024), differing training algorithms from linear parametric to nonlinear parametric, fitting GEI terms (Adunola et al., 2023; Brault et al., 2021; Jung et al., 2022; Nascimento et al., 2024), fitting inbreeding coefficients (Minamikawa et al., 2024), multivariate modeling (Brault et al., 2021; Jung et al., 2022), and even stacking ensemble learning (Nascimento et al., 2024).

Although PS has only recently been adopted in perennial crops, it remains true to its original goal of cost‐effectiveness, with numerous studies employing unmanned aerial vehicle‐ or laboratory‐based multispectral and hyperspectral sensors for its application. PS has proven useful across a range of perennial species, such as coffee (Adunola, Tavares Flores, Riva‐Souza, et al., 2024; Mbebi et al., 2022), alfalfa (Biswas et al., 2021), slash pine (Y. Li et al., 2023), and eucalyptus (Mieres‐Castro et al., 2024; Mora‐Poblete et al., 2024) for predicting yield, biomass, and growth traits. Notably, in some cases like eucalyptus, the PS predictive ability even exceeded that of GS (Mora‐Poblete et al., 2024). However, PS predictive ability can vary by species and trait, as illustrated by lower performance in certain apple fruit quality traits (Jung, Hodel, et al., 2025) and for malic and tartaric acids in grapevine (Brault et al., 2022). Yet for agronomic traits in grapevine related to cluster architecture, PS and GS provided similar predictive ability. For both PS and GS approaches applied in perennials, there is potential for enhancing predictive ability with the majority of studies reporting low to moderate correlations. There are common themes between both approaches in perennials that likely contribute to this observed lower predictive ability.

Several studies in perennial crops have investigated whether factors such as molecular marker density, marker type, or the genomic area covered (e.g., coding vs. noncoding regions) impact the predictive ability of GS. For instance, one apple study showed no significant difference in prediction accuracy between using 303,239 SNP markers and 10,295 SNPs (Cazenave et al., 2022), and similar results were seen in grapevine (Flutre et al., 2022). Conversely, expanding marker coverage in apple, especially into noncoding regions, did increase predictive ability for some quality traits (Minamikawa et al., 2024). These findings highlight that outcomes are often study‐specific. Higher marker coverage could be advantageous in populations with rapid linkage disequilibrium decay, a known characteristic of highly heterozygous outcrossing species like apple and many other perennials (Bianco et al., 2014). Consequently, the optimal marker density for GS depends on context, likely including TP composition and size. For PS, the inherent complexity in optimizing the density and combination of phenomic features presents a significant challenge and necessitates further research. Compounding this, the actual effectiveness of any chosen PS approach in practice is highly contingent on the specific context, including the plant's phenological stage, its growth environment (glasshouse or field), the organ being assessed, and how these factors interact, thus understanding this overall effectiveness is key to guiding truly practical and cost‐efficient implementation.

A common observation across GS studies in perennials is that traits with a low broad‐sense heritability have a low predictive ability (Arojju et al., 2020; Brault et al., 2021; Malmberg et al., 2023; Muranty et al., 2015). This finding is also commonly observed in other crop species (Voss‐Fels et al., 2019), highlighting the importance of using high‐quality phenotypic data to train predictive models. High‐quality phenotype characterization is essential because it ensures that the true genetic variation underlying complex traits is accurately captured. Crossa et al. (2017) and Resende et al. (2012) reinforce this essential requirement in cereals and perennials, respectively, and reason that the complex GEI present in crops further necessitates the requirement for high‐quality data. Furthermore, large TP sizes with phenotypes sampled across several years and environments is one possible approach to achieving high‐quality phenotype data, yet this is a common challenge for perennial crops.

Previous GS and PS reports in perennials typically have small TP sizes, often ranging from 200 to 500 individuals. Yet, it is well established that the accuracy of GS increases with the size of the TP, particularly for traits with low heritability (Daetwyler et al., 2012). Small populations sizes are a persistent challenge in perennials, driven by several features unique to this class of crop species. The long generation time and clonal propagation common to perennial crops delay the capture of reliable phenotypic data. Moreover, their regenerative nature and extended lifespans, combined with environmental variability and GEI, necessitate repeated phenotyping over multiple years. Considering these crops often require extensive land for field trailing and the prolonged duration (e.g., at least a decade), the cost of managing and phenotyping field trials vastly outweighs that of annual species.

In perennials, progress in prediction algorithms has generally not resulted in substantial improvements in the model's predictive ability, suggesting that the choice of model is likely to have a marginal impact on overall performance. Recently, Nascimento et al. (2024) tested the effect of fitting a genomic best linear unbiased prediction (GBLUP), a multivariate adaptive regression spline and a random forest model in their prediction framework for coffee, and found no significant differences in accuracy across the majority of traits. The authors did observe, however, that as the complexity of the trait genetic architecture increased and so with it small model differences did emerge, yet none were significant. Interestingly, gains in prediction accuracy of almost 200% (relative to GBLUP) were achieved when the best stacking ensemble approach was adopted (Nascimento et al., 2024). However, the extent of accuracy improvement varied among traits, likely due to differences in their genetic architecture. Similar increases in accuracy have been reported for ensemble approaches in cereals (Azodi et al., 2019). The ensemble approach is a relatively newly developed concept and demonstrates promise; however, high computational demands, especially in a GEI modeling prediction framework, have been identified as potential limitations. While within the PS framework, one study suggests that the model chosen may impact predictive ability, whereby deep learning methods outperformed Bayesian approaches in eucalyptus for the prediction of growth and wood quality traits. In this same study, the combination of PS with SNP marker and haplotype information enhanced prediction accuracy, especially for complex traits such as foliar oil content and stem volume (Mora‐Poblete et al., 2024). Although initial research outcomes appear promising, the adoption of PS in perennials is still recent, and thus further research is required to determine the impact of model complexity on predictive ability.

Many perennial crops are highly heterozygous due to their predominantly outcrossing nature, in contrast to major annual crop species, which are often inbred. Despite this, most GS models applied in perennials assume purely additive genetic architecture, potentially overlooking important sources of genetic variation. To better capture this variation, the inclusion of nonadditive genetic effects such as dominance and epistasis warrants further exploration. A study in coffee explored the impact of fitting relationship matrices that capture statistical dominance and epistasis and found a significant prediction gain compared to the purely additive model (Coelho de Sousa et al., 2022). However, a recent study in apple by Jung, Quesada‐Traver, et al. (2025) found that incorporating dominance effects using orthogonal variance partitioning did not meaningfully improve predictive ability and, in some cases, reduced the total explained variance. These findings highlight that while nonadditive effects appear biologically relevant in perennial crops, their utility in GS remains context‐dependent and requires careful modeling and validation. Similarly, cereal crops models incorporating nonadditive effects, like epistasis, have on occasion demonstrated improved prediction accuracies compared with purely additive models (Schrauf et al., 2020). However, other studies have reported no effect (Jarquín et al., 2014) or a negative effect (Lorenzana & Bernardo, 2009) of modeling epistasis on prediction accuracy.

### 2.2 Single‐omics prediction in perennial crops integrating GEI

Phenotypes emerge from the interplay between genotype and environment, making the prediction of genetic merit across diverse growth conditions challenging due to GEI. These interactions occur primarily in two forms: scale GEI, where relative genotype performance remains consistent across environments but with varying magnitudes of phenotypic differences due to environmental effects, and cross‐over GEI, where the performance ranking of genotypes reverses between environments, highlighting the complex nature of adaptation and phenotypic plasticity. Modeling the nuances of GEI is highly beneficial for crop genetic improvement programs, as it fulfills multiple objectives, such as (i) characterizing GEI to define target population environments (TPE), (ii) enhancing prediction accuracy across diverse environments, and (iii) providing new insights into the biological features underlying GEI, that collectively lead to improved genetic gain. By characterizing the GEI across a breeding program using GEI modeling approaches, the TPE can likely be better defined with the use of relevant latent model and environmental variables. Prediction models can focus then on single TPEs, and as a result, subsequently disentangle the cross‐over GEI to improve the prediction accuracy and allow breeders to increase their selection intensity within that specific TPE. Characterizing GEI can further provide insight into the genetic mechanisms underlying genotype stability across environments (e.g., across TPE), and thus allow more accurate prediction of stability. Taken together, breeders can use these improved predictions of performance within and across TPEs to design and optimize their selection strategy to breed for genotype stability and/or environmental specificity. A limitation of this approach, when using latent statistical modeling, is the limited biological insight into the mechanisms influencing these GEI and the environmental variables driving them. However, there is potential to overcome these limitations with the integration of crop growth models (e.g., Agricultural Production Systems sIMulator, Crop Environment Resource Synthesis, and the Decision Support System for Agrotechnology Transfer) and radiative transfer models (e.g., PROSPECT, SAIL, and their combined form, PROSAIL), which are mechanistic in nature, therefore having biological relevance when combined with environmental variables in the GEI modeling. Outcomes from this modeling could provide a deeper understanding of the adaptive genetic mechanism (e.g., genes and pathways) that drives stability and enables plants to respond to varying environmental stresses, as has already been demonstrated for shoot branching in plants using a gene‐to‐phenotype network (Powell et al., 2022). In addition, GEI modeling could reveal key genes and mechanistic pathways that contribute to phenotypic plasticity that allows the same genotype to adapt their physiology, morphology, and metabolism to different environmental conditions. Although not mutually exclusive, the evolution of such adaptation and plasticity mechanisms could be explored within breeding programs and more broadly in classes of species.

An appropriate modeling of GEI, particularly the genotype‐by‐year component, is crucial for accurately predicting genetic effects in perennial crops due to their multi‐season regenerative growth and exposure to diverse environmental conditions over their long lifespans. While several studies in crops like grapevine, apple, coffee, and perennial ryegrass have incorporated basic GEI terms into models (e.g., Adunola et al., 2023; Brault et al., 2021, 2024; Cazenave et al., 2022; Jung et al., 2022; Jung, Quesada‐Traver, et al., 2025; Pembleton et al., 2018; Sousa et al., 2019), these approaches often consider environments as independent, which may limit their ability to capture the complex covariance of genotype performance across environments and leaving GEI variance unaccounted for. Consequently, GEI remains underexplored within many perennials, especially compared to annual crops where advanced GEI modeling approaches that account for covariance are more routinely applied and have shown clear benefits (e.g., Tolhurst et al., 2022). A notable exception in perennials is a study using a factor analytic (FA) model in ryegrass, which successfully identified complex GEI patterns and redefined mega‐environments for breeding (Zhu et al., 2023). Further research implementing and evaluating such more advanced GEI models in diverse perennial systems is needed.

This GEI modeling study in perennial ryegrass highlights the utility of statistical approaches to characterize GEI and define TPEs, revealing discrepancies between the GEI patterns in latent modeling and historically defined agroecological zones. While latent statistical models provide valuable insights, their interpretability is limited as the direct environmental parameters that affect the genotype's performance remain elusive. To address this limitation, alternative approaches like extended Finlay Wilkinson regression with the integration of multiple environmental covariates (Piepho & Blancon, 2023) and the integrated factor analytic linear mixed model (IFA‐LMM; Tolhurst et al., 2022) have been proposed. Multivariate regression approaches (e.g., Finlay Wilkinson) model the relationship between predictors (e.g., weather variables) and responses (e.g., phenotype) by reducing the predictors into orthogonal components that maximize covariance with the response, making them well‐suited for high‐dimensional and collinear data. The latter models can also include residual FA variance–covariance structure in order to incorporate latent variables (Piepho & Williams, 2024). Similarly, the IFA‐LMM integrates latent factors and explicit environmental covariates within the FA framework, and when applied in cotton, the IFA‐LMM enabled GEI interpretability and increased predictive ability (Tolhurst et al., 2022). Being hierarchical in nature, the two latter approaches have the flexibility to be extended into a multi‐omic framework.

A potential limitation of approaches like the IFA‐LMM is their linear nature, which may have limitations in capturing the complex nonlinear interactions likely present in multi‐omics data. As an alternative, Costa‐Neto et al. (2021) demonstrated the potential of the nonlinear hierarchical Bayesian models, which integrate GEI, dominance effects, and environmental covariates, achieving superior predictive ability in maize datasets compared to traditional GBLUP models. Additionally, the crop growth model–whole genome prediction (CGM–WGP) approach that also uses a hierarchical Bayesian model to capture GEI could be a suitable alternative and has been demonstrated to increase predictive ability in maize (Cooper et al., 2016; Messina et al., 2018; Technow et al., 2015). The CGM–WGP approach integrates mechanistic crop growth models with genomic data to capture dynamic physiological responses as well as nonlinear interactions, and thereby likely nonadditive genetic effects, potentially providing deeper insights into key biological components of GEI, such as stress responses, metabolic regulation, and growth dynamics. While these modeling approaches show great potential for capturing the high complexity and dimensionality of GEI in perennials, they have yet to be widely adopted, and would also likely provide valuable insights into long‐term GEI patterns, perennial adaptation, plasticity, and potentially even cross‐cycle influences, for example, linked to epigenetic effects.

### 2.3 Benchmarking the efficacy of single‐omic prediction frameworks

Rincent et al. (2018) originally introduced PS as a potential alternative to GS, particularly in resource‐limited contexts, and as such the majority of literature in perennials has compared the utility of PS by benchmarking the predictive ability of PS against GS. In grapevine, the study by Brault et al. (2022) is a prominent example, where the predictive ability of PS and GS was compared in a panel of varieties and a biparental population for several agronomic and quality traits. The authors observed comparable performance between PS and GS for cluster architecture traits, but GS demonstrated superior accuracy for quality traits like malic and tartaric acids. Similarly, Adunola et al. (2024) benchmark PS against GS for prediction of yield in *Coffea canephora* and show promising results for the use of near‐infrared reflectance (NIR) on green coffee beans for yield prediction. The authors highlight key nuances in the application of PS, first the inability to disentangle the additive genetic component of the prediction, a critical consideration when using the predictions for parental selection, and second the risk of bias toward the specific environmental conditions under which the phenomic data were collected. There are ways to circumvent these challenges; however, the influence and potential bias of the environment raises an important difference between PS and GS, in that genomic data can be considered static and typically not confounded with environmental effects, whereas PS is likely substantially more affected by such influences.

This key differential in the approaches directly affects then the relationship between predictive ability and prediction accuracy for each approach. Herein this review we refer to *predictive ability* as the Pearson correlation coefficient between estimated breeding values and observed phenotypes, while *prediction accuracy* refers to the correlation between the estimated and true breeding values (Legarra et al., 2008). F. Wang et al. (2025) highlight that while predictive ability always underestimates accuracy for additive GS in a consistent and correctable way (e.g., by dividing by the square root of the narrow‐sense heritability), it may over‐ or underestimate accuracy in PS depending on the extent to which phenomic features are correlated with nonadditive genetic effects or environmental noise. Despite being the norm, this makes direct comparison between PS and GS models using cross‐validation (CV) correlations that typically use predictive ability inherently unreliable. To address this, F. Wang et al. (2025) propose a more robust alternative to correct the predictive ability of PS models using a genetic correlation‐based approached, which estimates prediction accuracy as the genetic correlation between predicted and observed phenotypes scaled by the heritability of the predicted phenotypes. Through the use of three worked examples using published data in wheat and poplar (Krause et al., 2019; Rincent et al., 2018; Winn et al., 2023), the authors demonstrate that following an appropriate correction, the prediction accuracy of GS and PS models are directly comparable, and that GS consistently yielded significantly higher prediction accuracies than PS. Even when prediction accuracy is appropriately calculated, F. Wang et al. (2025) emphasize that this metric alone is insufficient to evaluate the utility of PS versus GS, a perspective that is emerging in recent perennial studies. The rationale is that prediction accuracy is just one element determining genetic gain according to the breeder's equation. Since PS and GS may uniquely affect the equation's other variables, evaluating their comparative utility requires assessing their total potential contribution to overall genetic gain.

Evaluating breeding technologies requires looking beyond just prediction accuracy, as shown by Adunola, Tavares Flores, Azevedo, et al. (2024) in a blueberry program comparing PS and GS. They considered not only accuracy but also how the timing of selection (affecting cycle length) and the tissue measured (leaves vs. fruit) impacted overall genetic gain. Their findings reveal how the strategic application of these technologies matters greatly. For example, PS applied early to leaf tissue for total titratable acidity, despite modest accuracy (*r* = 0.36), resulted in a high relative increased genetic gain (30.2%) due to faster cycling, surpassing later fruit‐based PS and nearly equaling GS. However, for firmness, GS provided the best outcome (9% relative gain) because its higher prediction accuracy was combined with the ability to select early at the seedling stage. Thus, the study illustrates that the most effective approach can depend heavily on when in the breeding cycle it enables decisions, making these practical factors key determinants of genetic gain alongside prediction accuracy itself.

While Adunola, Tavares Flores, Azevedo, et al. (2024) maintained a constant selection intensity in the analysis of their study, it is important to note that the highly cost‐effective nature of PS lends the approach to larger population sizes, compared to GS models, and thus a potential increase in selection intensity. However, as Cobb et al. (2019) highlighted, the relationship between population size and genetic gain is not linear. Nonetheless, it is apparent that both technologies differentially affect components of the breeder's equation, thus any meaningful comparison of GS and PS should move beyond pure predictive ability and instead quantify their impact holistically as net impact on genetic gain per unit time. In this context, the integration of phenomic and genomic data within a multi‐omics prediction framework and any further omic extensions inherits many of the same challenges identified for PS alone, and these nuances must be carefully addressed in future benchmarking to ensure fair and meaningful comparisons with GS models.

### 2.4 Multi‐omic prediction in perennial crops

Multi‐omics‐based prediction models for perennials are still in their infancy, with five foundational studies to date covering grapevine (Brault et al., 2022), perennial ryegrass (Malinowska et al., 2022), coffee (Adunola, Tavares Flores, Riva‐Souza, et al., 2024), blueberry (Adunola, Tavares Flores, Azevedo, et al., 2024), and apple (Jung, Hodel, et al., 2025). In grapevine, GS and PS were combined within a diverse population to explore predictive ability for agronomic and quality traits (Brault et al., 2022). The authors found no significant increase in predictive ability for the multi‐omics approach, above that of PS and GS, in either trait categories. Similarly, using an apple reference population and related breeding material, the integration of genomic and phenomic data in an extended GBLUP approach, Jung, Hodel, et al. (2025) demonstrated that for most traits GS and multi‐omic models performed similarly, while PS performed substantially worse. However, both studies employed a relatively simple GEI model, and it is likely that a portion of the GEI variance was not fully captured and instead remained in the residual error term, potentially underestimating the contribution of environment‐specific effects, particularly relevant for phenomic data. Furthermore, the phenomics data used in the apple PS were collected at a single time point, and it is highly likely that the developmental stage at which the data are captured plays a substantial role in predictive ability, where dynamic physiological changes across the season can significantly influence the spectral signal. As such, capturing phenomic features at only one growth stage likely limited the ability of the phenomic models to reflect future performance, restricting them instead to traits already expressed at the time of sampling. Therefore, future work is needed to better understand trait dynamics across development and identify optimal temporal strategies for PS data collection. Similarly, in blueberry, multi‐omics models that integrated genomic and phenomic data in a Bayesian regression approach showed moderate, trait‐ and tissue‐dependent improvements, but these gains over GS or PS models alone were not consistent (Adunola, Tavares Flores, Azevedo, et al., 2024). In contrast, Adunola, Tavares Flores, Riva‐Souza, et al. (2024) found that combining genomic and phenomic data in a multi‐kernel mixed model consistently improved predictive ability over GS alone, indicating that the phenomic features captured complementary information valuable for prediction. This result in coffee may stem from the use of phenomic features captured on green beans, tissue closely linked to the target trait of yield, combined with careful standardization and trait‐specific spectral patterns that capture complementary genetic variance to genomic data. While providing important foundational insights, the studies by Brault et al. (2022), Adunola, Tavares Flores, Riva‐Souza, et al. (2024), Adunola, Tavares Flores, Azevedo, et al. (2024), and Jung, Hodel, et al. (2025) collectively highlight that the value of multi‐omics models is highly context‐dependent, shaped by factors such as tissue type, trait architecture, and the degree of complementarity between data sources, underscoring the need for further research to optimize their implementation across diverse crop species and breeding objectives.

Beyond PS and GS, a study in perennial ryegrass demonstrated that incorporating additional omics data in the form of transcriptomic and epigenomic profiles allowed a multi‐omics model to explain a greater proportion of the total phenotypic variance, reflecting improved capture of the variance components attributable to genetic effects, GEI, and environmental interactions (Malinowska et al., 2022). Although the authors did not explicitly calculate predictive ability, the increased capture of and improved partitioning of variance indicate likely model improvements. Multi‐omics prediction appears promising for perennials, but its adoption has been slower than in annuals. This lag may in part be due to differences in funding structures, while breeding in annual crops is often supported by commercial investment, perennial breeding typically takes place within publicly funded institutions with more limited resources.

The potential for multi‐omic prediction models in annual crops have been described in maize (Wu et al., 2024), rice (Arouisse et al., 2021; Matsuda et al., 2015; S. Wang et al., 2019), wheat (Crain et al., 2018; Krause et al., 2019), cotton (M. Wang et al., 2016), and potato (D. Li et al., 2024). Specifically, in maize for grain yield, the effective integrations of genomic, phenomic, and metabolic data in a machine learning approach achieved over 10% gain in prediction accuracy compared to any single‐omic prediction model, where several models across three classes (e.g., linear regression–based, tree‐based, and neural network–based models) were compared (Wu et al., 2024). Beyond potentially improving prediction accuracy, multi‐omics models have the potential to reveal biological mechanisms that may seem unattainable through single‐omic approaches, as exemplified by Wu et al. (2024) identifying regulatory pathways for maize photosynthesis and kernel development linked to source strength and yield, providing valuable insights for precision breeding approaches.

Machine learning models have commonly been applied in several annual crop multi‐omics prediction frameworks, and are likely well suited as they are highly flexible in their prediction tasks and can capture and respond to complex, nonlinear interactions within and between diverse biological layers. A recent study in maize demonstrates that the use of machine learning models for prediction in a large‐scale breeding population outperformed the other multi‐omic prediction models (e.g. GBLUP, support vector regression, deep GS; K. Wang et al., 2023). The prediction accuracy improvements ranged from 50.9% to 167.0%, yet most importantly the neural network maintained high predictive ability even for small datasets, underscoring its versatility and potential for diverse breeding applications in perennial crops. While the neural network was able to maintain predictive ability for a small TP in the latter scenario, population size is still likely a major bottleneck in realizing the advantages of machine learning approaches.

In the context of alternative multi‐omic prediction methodology, Christensen et al. (2021) and Zhao et al. (2022) propose an approach that extends on linear mixed model into multilayer neural networks to integrate multi‐omic data. These multilayered neural networks are proposed to capture the nonlinear relationships between the omics features and the phenotype, and the authors use the same simulated data set to explore predictive ability and assess the impact of incomplete omics data, thereby evaluating the robustness of their models in scenarios where certain omics measurements are missing. In the base‐line model with fully observed datasets, Christensen et al. (2021) demonstrate an increase in predictive ability for the multi‐omics BLUP model (*r* = 0.79) compared to pedigree BLUP (*r* = 0.43) and GBLUP (*r* = 0.70). The authors liken the impact of including omics data to that of including correlated traits, and note that the increase in predictive ability is dependent on the strength of the relationship between the phenotype and the omics features as well as their heritabilities. In this same study, the incomplete omics dataset yielded comparable predictive ability, and Zhao et al. (2022) further enhanced these predictions by employing advanced modeling approaches specifically designed to mitigate data sparsity. Although both authors acknowledge limitations, such as using a single simulated dataset and with high heritability estimates for traits and omics features, their promising methodological frameworks are further limited by the omission of GEI. Predictive modeling in crops is advancing; however, no existing multi‐omics frameworks seems capable of fully capturing whole system complexities. In contrast, human genetics has used deep learning for multi‐omics prediction, notably through convolutional neural networks in combination with methods like DeepInsight (Sharma et al., 2019), which transform nonimage data into image‐like representations for improved latent feature extraction. Despite these advancements, challenges persist, including model interpretability, data heterogeneity, and appropriate management of large datasets (Sharma et al., 2024).

## 3 INTEGRATING MULTI‐OMICS PREDICTION INTO BREEDING PRACTICE

**FIGURE 2:**
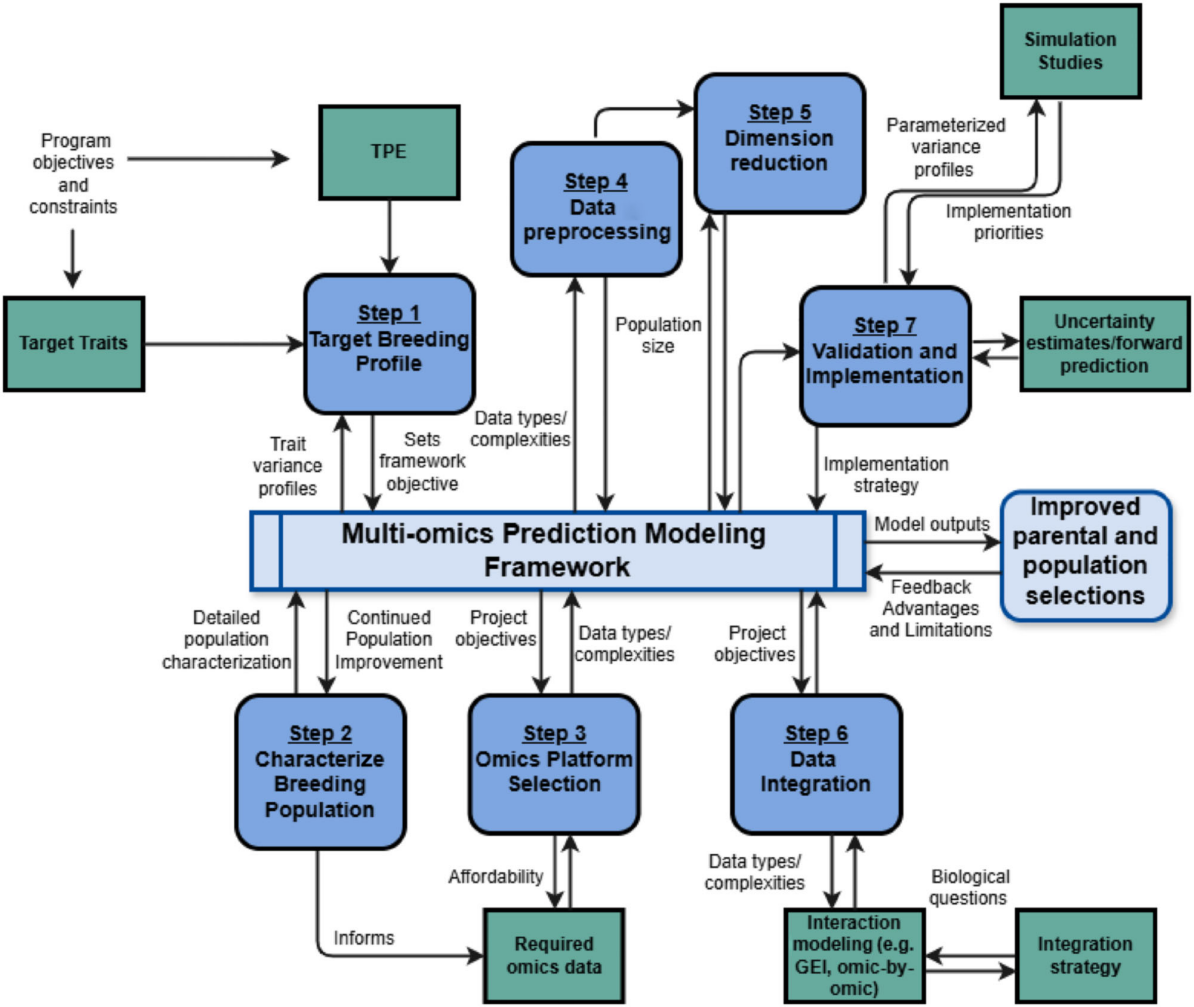
A conceptual framework outlining the necessary steps for developing, validating, and implementing multi‐omics prediction models to enhance selection decisions in perennial crop breeding programs. The blue rounded boxes represent the core process steps, while the teal square boxes describe key inputs, outputs, and considerations for the respective process. TPE, target population environments; GEI, genotype‐by‐environment interaction.

Multi‐omics prediction modeling promises enhanced accuracy, and to gain deeper biological insights into complex features such as GEI, but realizing its practical potential in perennial breeding requires addressing inherent challenges. Key open questions persist around optimizing data preprocessing, integration frameworks, and appropriate modeling techniques to effectively manage complex spatial‐temporal features and data heterogeneity. Herein, we outline a conceptual structured framework (Figure 2) to guide the application of multi‐omics prediction within perennial crop breeding programs, encompassing key stages from initial objective setting through to practical implementation, while addressing associated challenges and opportunities.

### 3.1 Framework for multi‐omics prediction in perennial breeding

As foundational to any effective breeding strategy, the framework commences with establishing a clearly defined and achievable target breeding profile (Figure 2, Step 1). This profile must articulate priority breeding traits, ensuring they align with program goals, budget constraints, and are realistically achievable based on evidence of sufficient genetic variance within the breeding population. Concurrently, the profile requires a well‐characterized TPE, typically delineated through robust multi‐environment trial analysis with appropriate GEI modeling. Integral to defining these targets and identifying opportunities for continual improvement is an in‐depth characterization of the breeding population itself, encompassing its genetic structure, signatures of selection, and the architecture of key traits (Figure 2, Step 2). Establishing these strategic foundations is prerequisite to the subsequent step of generating informative multi‐omics data to drive parental selection and accelerate population improvement.

In the context of omics data availability, GS and PS are prevalent in current predictive breeding research studies, likely due to their accessibility. However, incorporating alternative endophenotypic data, such as epigenetic, transcriptomic, and proteomic information, into predictive breeding frameworks remains minimal, and historically largely a result of the cost‐prohibitive nature of generating these types of data. Yet, as technologies advance and data generation costs decrease, large‐scale endophyte data will likely become more accessible. However, key considerations remain and include whether omic technologies can be made more cost‐effective per sample, whether different omics resources can be simultaneously generated to improve affordability, and how to determine the most relevant technology platforms for specific breeding objectives (Figure 2, Step 3). To illustrate potential opportunities, recent sequencing technologies, such as Oxford Nanopore and Pacific Biosciences, have simplified and enhanced the sequencing process by simultaneously generating genomic and epigenomic information. By analyzing native DNA modifications, these methods eliminate the need for additional experiments, enabling the direct assessment of genetic variants and methylation status across the entire genome. Besides providing obvious benefits in understanding epigenetic causes for phenotypic variation, this technology advancement substantially decreases costs, increasing accessibility to epigenomic data. As an example of the utility of these data, evidence exists for the role of epigenetic modifications in adapting grapevines to different environments through gene regulation in response to environmental stimuli (Xie et al., 2017). Therefore, the generation of high‐throughput data could support large‐scale assessments of genetic and epigenetic diversity in large perennial crop populations, and its value has already been demonstrated in dairy cattle (González‐Recio et al., 2023). While similar studies in perennial crops have yet to be published, early research strongly suggests that epigenetic mechanisms play a key role in complex trait variation (as reviewed by Berger et al., 2023 for grapevine). Leveraging this knowledge could enhance predictive ability, especially when integrated with multi‐omic datasets.

### 3.2 Curation, integration, and modeling for high‐dimensional omic data

When considering the integration of multiple omic data sources it is important to recognize that the data are inherently intricate, highly heterogenous, and multidimensional, encompassing diverse layers of biological information spanning different spatial (e.g., from cells to canopies), temporal (e.g., milliseconds to years), and measurement modalities (e.g., technologies utilizing X‐rays vs. infrared). Effective data integration requires appropriate statistical models to reduce noise, adequately capture relevant relationships, and extract meaningful and ideally interpretable patterns. Yet prior to such integration, and arguably of equal importance, is data preprocessing, which should include rigorous quality assurance and quality control for data curation, vital to the downstream interpretation and utility (Figure 2, Step 4). Hyperspectral data are a good example where preprocessing eliminates spectral artefacts and addresses issues in image segmentation, correction, and space‐spectral dimensional adjustments, typically through dimension reduction, resolution enhancement, and geometric and spectral corrections (Cozzolino et al., 2023). Each of the latter preprocessing components are highly important (as reviewed by Aspinall et al., 2002 and Burger & Gowen, 2011); however, the spectral correction step is crucial for minimizing noise and enhancing reflectance differences between wavelengths, and subsequently genotypes. The Savitzky–Golay filtering and the continuum removal methods are both examples of widely adopted and effective preprocessing approaches to spectral correction in hyperspectral data (Kale et al., 2017; Thorp, 2024). While this is just one example, it is essential to account for the unique preprocessing requirements of each omics dataset, alongside its spatiotemporal context, before integrating it into the multi‐omics framework.

To be effective, the integration approach must handle the extensive dimensionality and interdependencies of the data outlined above. For perennial crops, which often have smaller population sizes, the high‐dimensionality of omics data presents additional challenges. Data reduction techniques, such as principal component analysis or factor analysis, prior to integration of omics data can help manage these complexities but require careful consideration to avoid losing biologically relevant information (Figure 2, Step 5).

The goal for Step 6 in the framework is the critical task of combining diverse data layers to capture biological complexity. Two broad data integration strategies have previously been defined, these being multistaged and meta‐dimensional analyses (Ritchie et al., 2015), each offering unique advantages and challenges. Multistaged analysis deals with high data dimensionality by implementing a linear, hierarchical stepwise approach to minimize the search space. At each step, only two datasets are analyzed at one time (e.g., SNP markers and phenotypes), and at each successive step the genetic signal is enriched by the next association analysis (e.g., SNP markers and epigenomic profiles). A well described example of this is the triangle method used to identify functional SNP marker–trait associations (Holzinger & Ritchie, 2012). While computationally efficient, a clear limitation of this approach is the stringent boundaries pertaining to the analysis of only two variables at a single step, thus limiting the ability to detect variance that is a product of several interacting omic layers.

Meta‐dimensional analysis overcomes limitations of multistaged approaches by simultaneously analyzing interactions across biological scales, using strategies categorized as concatenation‐based, transformation‐based, or model‐based integration (Ritchie et al., 2015). The latter, which synthesizes outcomes from individual data‐type models, holds particular promise for tackling complex biological phenomena like GEIs. Integrating multi‐omics data into GEI modeling aims to capture nonlinear interactions and nonadditive effects, thereby enhancing prediction accuracy and providing biological insight into processes, for example, underpinning stress response and metabolic regulation. Advanced techniques are required for this, such as the previously described model‐based approach combining linear mixed models and multilayer artificial neural networks (ANNs), demonstrated by Christensen et al. (2021). In their study, they successfully applied this hybrid framework to simulated multi‐omic data, demonstrating its potential to improve prediction accuracy for complex traits by effectively capturing interaction effects, even under sparse data conditions. Alongside ANNs, the previously described CGM–WGP framework represents another promising avenue. However, the integration of robust GEI modeling within multi‐omics prediction frameworks is still at an early stage in crop research, highlighting a promising gap for further development. Future work should prioritize applying and evaluating these advanced methods (ANNs, CGM–WGP, etc.) also in perennial breeding contexts, either directly or via realistic simulations ideally informed by real‐world data, to support realizing their full potential for crop improvement.

In perennial breeding programs, typically focused on crossing highly heterozygous and in many cases interspecific hybrids, both additive and nonadditive genetic effects are likely very important for both parental selection and variety development. Recent studies have underscored the importance of considering nonadditive effects when selecting parents based on genomic predictions of progeny performance in clonal breeding programs (Werner et al., 2023). However, minimal previous research has explored optimizing parental selection in perennials, particularly with regard to the significance of nonadditive genetic effects. In sugarcane, Yadav et al. (2023) demonstrated that mate‐allocation strategies based on clonal performance (including nonadditive genetic effects) resulted in substantial improvements in predicted progeny values, ranging from 16% to 57% for yield and quality traits, thereby highlighting the potential of the proposed approach for practical breeding applications. Within this context, multi‐omics prediction offers the potential to capture nonadditive effects more effectively by modeling the multilayered interactions among omics features, the environment, and the target phenotype(s).

### 3.3 Model validation and practical implementation

Rigorous model validation is paramount to demonstrate the practical utility of any multi‐omics prediction framework and build confidence in its application for perennial breeding (Figure 2, Step 7). This necessitates not only employing appropriate model comparison statistics but also utilizing approaches capable of quantifying prediction uncertainty across influential factors, including genotypes, latent variables derived from omics data and the environment, along with environmental covariates. Central to robust validation are appropriate CV strategies. Thus far, validation in perennial predictive modeling, encompassing both single‐ and multi‐omic approaches, has predominantly relied on random CV or leave‐one‐environment‐out schemes. While some research, such as Jung, Hodel, et al. (2025) in apple, has explored predicting into genetically unrelated material, a critical gap remains in assessing true forward prediction performance, specifically, forecasting the performance of novel genotypes in unobserved future environments. A notable example is in strawberry, where forward prediction was successfully used to assess the utility of GS for parental selection across breeding cycles (Osorio et al., 2021), aligning model evaluation with real‐world application. Evaluating models under such demanding forward prediction conditions is essential for truly assessing their potential utility, yet practical implementation faces further hurdles, particularly concerning resource limitations common in perennial breeding programs.

Given the constraint of limited resources in perennial breeding it is impractical to empirically test every new predictive breeding technology and optimize its deployment at each breeding stage. However, without such optimization, even promising new technologies may fail to deliver practical benefits. Breeding program simulation studies offer valuable insights into the benefits and challenges of applying multi‐omics prediction frameworks by constructing extensive TP and examining the impact of the approach on each component of the breeder's equation, ultimately facilitating the effective integration into breeding practices. High‐quality simulation tools, such as MoPS (Pook et al., 2020), AlphaSim‐R (Gaynor et al., 2021), and genomicSimulation (Villiers et al., 2024), allow these simulations to be parameterized with real breeding program features and model parameters (e.g., variances and heritabilities) derived from prior empirical studies. These parameters can then be used to expand simulation scenarios beyond what is empirically achievable. For instance, Papin et al. (2024) combined empirical data with extended stochastic simulations to assess the accuracy of GBLUP for both global and within‐family predictions in conifers, and demonstrated across both scenarios that TP size critically influences prediction accuracy. These results highlight the huge potential of simulation studies in optimizing the application of predictive breeding technologies, particularly in perennial crop breeding context where breeding cycles are typically extremely long. They also emphasize the need for further research, particularly through direct application in perennial systems or simulations tailored to the unique characteristics of perennial crop breeding, to fully harness the potential of multi‐omics prediction approaches.

While optimizing the affordability and practical application of omic technologies is critical, their impact on predictive ability will remain constrained unless the challenge of small TPs in perennials is addressed. The predictive ability of GS models is fundamentally linked to the size and composition of the TP (Daetwyler et al., 2012). Given the absence of large commercial improvement programs for many important perennial crops, a promising solution involves cross‐institutional collaboration to pool standardized phenotypic, genotypic, and potentially multi‐omic datasets, thereby creating larger, shared reference populations that enable substantially increased predictive power. This strategy has proven highly effective in human genetics (e.g., biobank resource; Bycroft et al., 2018) and animal breeding, particularly dairy cattle, providing a strong proof‐of‐concept for increasing GS accuracy through reference populations exceeding 1.3 million individuals (Wiggans et al., 2017). Although perhaps less formally established as a widespread, standalone strategy in plants compared to livestock, the principles underpin successful collaborative breeding initiatives where shared data resources enhance breeding outcomes. In practice for perennials, these shared datasets could be used as is or tailored by individual institutions, while ideally being maintained and updated collectively. While careful consideration must be given to population structure to avoid issues stemming from distant relatedness between the reference and selection candidates, such datasets can often be optimized at the institutional level. Moreover, even moderately related large TPs are likely to generate useful predictive ability for traits of moderate genetic complexity, such as the quality and disease resistance traits critical for many perennial fruit crops. Expanding this approach multinationally could further enhance its impact by enabling better characterization of GEI and delineation of TPEs across wider geographic regions, improving breeding precision. For perennial breeding programs facing resource constraints, collaborative TP approaches that learn from established systems present a compelling solution worthy of further investigation and adoption.

## 4 CONCLUSION

Many perennials are facing unprecedented challenges due to their high vulnerability to climate change and unsustainable production trajectories, necessitating urgent adaptation to the evolving agricultural landscape. Advanced predictive breeding technologies represent the most promising pathway for achieving rapid genetic improvement in these crops. Recent research in major annual crops demonstrates that by leveraging the integration of diverse omics resources and thus sampling multiple layers between the genome and the phenotype, there is immense potential for increasing predictive ability and accelerating genetic gain. Additionally, there is an opportunity to gain deeper insights into the complex biological interactions that regulate key traits, facilitating faster and more accurate recycling of parental lines and improving selection accuracy and intensity. To fully realize this potential, an end‐to‐end framework is essential for generating high‐quality, reproducible predictions; and such a framework must be flexible enough to capture additive and nonadditive genetic variation that is likely jointly comprised of omic‐by‐omic interactions and GEI. Moreover, to overcome resource constraints and the challenges of small population sizes for prediction model training in most perennials, it is critical to explore cross‐institutional collaborations that pool resources to design statistically powerful training data sets, and to use breeding program simulation tools to determine strategies for the seamless integration of multi‐omics‐based predictions into practical breeding operations. Realizing this potential will require substantial further research to refine data integration strategies, improve model interpretability, and ensure that multi‐omics‐based prediction approaches are effectively tailored to the unique biological and logistical challenges of perennial crops. Despite the long road ahead, multi‐omic predictive breeding holds great promise as a powerful breeding tool with the potential to accelerate genetic improvement in perennial crops in the future.

## AUTHOR CONTRIBUTIONS

**Hannah Robinson**: Conceptualization; investigation; writing—original draft; writing—review and editing. **Carlos A. Robles‐Zazueta**: Investigation; writing—original draft; writing—review and editing. **Kai P. Voss‐Fels**: Conceptualization; supervision; writing—original draft; writing—review and editing.

## CONFLICT OF INTEREST STATEMENT

The authors declare no conflicts of interest.
